# Prevalence and risk factors associated with gastrointestinal nematode infection in goats raised in Baybay city, Leyte, Philippines

**DOI:** 10.14202/vetworld.2016.728-734

**Published:** 2016-07-14

**Authors:** Ariel Paul M. Rupa, Harvie P. Portugaliza

**Affiliations:** Department of Veterinary Clinical Sciences, College of Veterinary Medicine, Visayas State University, Visca, Baybay City, Leyte 6521-A, Philippines

**Keywords:** goat, *Haemonchus*, Philippines, risk factors, strongyle, *Trichuris*

## Abstract

**Aim:**

Gastrointestinal parasitism is a serious constraint affecting goat production in the Philippines. This study aimed to determine the prevalence and associated risk factors of gastrointestinal nematode infection in goat-populated barangays of Baybay City, Leyte.

**Materials and Methods:**

A total of 81 households or farms were interviewed, and 450 goats were sampled for fecalysis. Fecal egg count along with egg morphological identification and coproculture for third stage larvae identification were conducted. Descriptive statistics and logistic regression analyses were carried out to determine the farm- and animal-level prevalence and risk factors.

**Results:**

Fecalysis revealed the presence of strongyle and *Trichuris* spp. with a farm-level prevalence of 100% and 4.94%, respectively; and animal-level prevalence of 96.22% and 4.44%, respectively. The identified strongyle genera per barangay were *Haemonchus* spp. (34.79%), *Trichostrongylus* spp. (33.29%), *Oesophagostomum* spp. (24.21%), *Cooperia* spp. (6.93%), and *Chabertia* spp. (0.79%). Goats older than 12 months were four times more likely to present high strongyle burden when compared to goats <6 months. With each month increase in goat’s age, the odds of acquiring strongyle infection also increased by 1.07 times. Animals kept in goat house with cemented flooring have lower odds of acquiring strongyle (odds ratio=0.12). Goats raised for leisure purposes and fed with carabao grass (*Paspalum conjugatum*) were 8.12 and 5.52 times more likely to acquire *Trichuris*, respectively.

**Conclusion:**

Most of the backyard goat raisers in Baybay City, Leyte, do not practice sound helminth control measures as shown by the high prevalence of gastrointestinal nematodes. The most relevant risk factors for gastrointestinal nematode infection were the age of the goat, type of goat house’s flooring, purpose of raising goats, and feeding practices.

## Introduction

Goat production holds an important niche for sustainable agriculture in developing countries and supports a variety of socioeconomic functions throughout the world. In Southeast Asia, small ruminants are the vital part in mixed farming systems adopted by smallholder farmers [1,2]. In fact, goat farming in the Philippines is considered the sunrise industry due to low capital investment and high rates of return [3] and thus provides livelihood to about 15 million Filipinos [4,5].

Livestock programs funded by the Philippines government targeting the remote rural areas of the country focused more on goat dispersal and production improvement to augment the income of many impoverish farmers. However, goat health problems remain the major impediment to goat production, suppressing the success of some government funded projects related to goat farming. Although mortality and morbidity rates in the herd vary depending on the causal agents, parasitism still ranks among the top three constraints in the Philippines [6,7].

The problems associated with gastrointestinal parasitism are often classified as production disease which results in feed intake reduction and alteration of gastrointestinal motility leading to diarrhea. Gastrointestinal parasites change the host metabolism accounting for much of the reduced protein and energy retention, and disturbed mineral and water balance [8]. Therefore, the economic losses are attributed to low production, high cost of prevention and treatment, and death of infected animals [1].

Studies dealing with the distribution and parasite control measures adopted by backyard goat raisers in different Philippines regions are very limited or absent, especially in remote rural villages of Visayas. Therefore, the present study aimed to identify the prevalence and risk factors associated with caprine gastrointestinal nematodes in goat-abundant barangays of Baybay City, Leyte. Knowing the prevalence and risk factors of parasitic nematodes is vital for future holistic prevention and control strategies in the area. The results gathered from this epidemiological investigation may also serve as a point of reference to other farms and villages with similar goat management and climatic condition.

## Materials and Methods

### Ethical approval

The study was conducted after the approval of the college research committee. A non-invasive fecal collection was performed by waiting for the animal to defecate. Therefore, handling was minimal and based on the guide for the care and use of agricultural animals in research [9].

### Description of the study area

**Figure-1:**
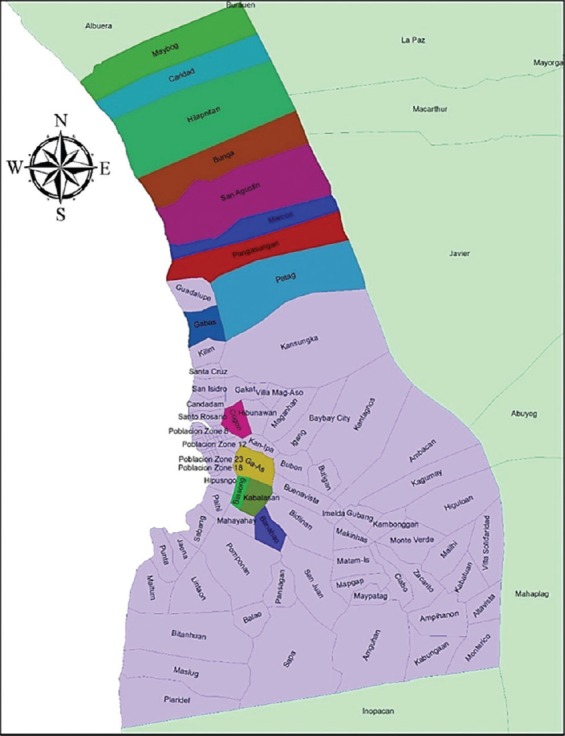
Map of the 14 sampled barangays in Baybay City, Leyte (Shapefile source: PHILGIS).

Baybay City (GPS coordinates: 10.6521412°N, 124.8525626°E) is located in the island of Leyte, Eastern Visayas, Philippines (Figure-1). The climate has no significant seasonal demarcation throughout the year and normally shows 80-90% relative humidity, 27.2-28.2°C ambient temperature, and 2500 mm average annual rainfall [10].

### Study design

A cross-sectional study was carried out in 14 villages or barangays identified by the Department of Agriculture (DA), Baybay City, with the highest number of goats. All 14 barangays were surveyed during the first quarter of the year 2016 to determine the number of farms or households raising a goat. A total of 81 farms/households were identified to have at least 1 goat. The sample size was then calculated using a 50% expected prevalence and 95% level of confidence interval (CI) with a 5% margin of error [11]; hence, the minimum sample size was 385. All 81 farms/households were interviewed, and a total of 450 goats were sampled for fecalysis. All goats in every herd present during the visit were sampled.

A structured survey questionnaire was formulated in English through Epi Info 7 and was translated into a local dialect during the interview. The questionnaire was designed to capture information related to epidemiological factors putatively associated with nematode infection; the intrinsic factors (i.e., animal signalment) and extrinsic factors (i.e., environment, feeding management, and the methods being practiced by the farmer in raising the goats).

### Fecalysis

Coinciding with the normal defecation time of goats from 5:30 am to 8:00 am, samples were collected by waiting for the feces to fall onto the gloved hand of the field collector. A palm full of fecal samples was collected in one goat. Samples were labeled accordingly and stored in ice chilled container to slow down the process of nematode eggs development during transportation. Determination of fecal egg per gram (EPG) count along with parasite genus based on egg morphology was performed according to Zajac and Conboy [12]. An aliquot of fecal mixture was filled into the chamber of Whitlock Universal slide and counted and calculated based on the formula:


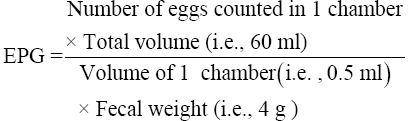


The technique has an analytical sensitivity of 30 EPG and specificity of identifying the eggs up to the genus level, except for strongyle. Therefore, strongyle genera were determined through third stage larvae (L3s) morphology [12].

### Identification of strongyle genus

Coprocultures of pooled fecal samples in 14 different barangays were performed to determine the distribution of different strongyles per village [13,14]. Macerated fecal sample (10 g) was loaded to a shot glass (height: 7.6 cm; diameter: 5.1 cm) with moistened cotton laden at the bottom. The shot glass was placed inside the culture glass (height: 10.2 cm; diameter: 7.6 cm) filled with water to the rim of the shot glass. The preparation was covered with carbon paper and stored in a room at 27°C ambient temperature for 7 days. After incubation, the water in the culture glass was examined for L3s. A total of 1400 L3s (i.e., 100 L3s per barangay) were collected in random and identified based on the identification key described by Zajac and Conboy [12].

### Data management and analysis

The data from the survey questionnaire were organized and cleaned using Microsoft Excel. Descriptive statistics and regression analyses were carried out to determine the farm- and animal-level prevalence and risk factors by using the program Epi Info version 7 (Centers for Disease Control and Prevention, USA). Based on EPG count, the degree of gastrointestinal nematode infection was classified into mild infection (EPG <500), moderate infection (EPG=500-1500), heavy infection (EPG=1501-3000), and fatal infection (EPG >3000) [15].

To assess the unconditional association between the presence of nematode (dependent variable) and the risk factors (independent variables) at the farm- and animal-level, a univariate and multivariate logistic regression analyses were performed. All independent variables that showed p<0.20 during the univariate analyses were included in a multivariate logistic regression model. A backward stepwise elimination approach was done with p<0.05 as the limit, removing the least significant variable one by one until the p value of the whole model was <0.05, and each remaining variable in the model had a p<0.05.

## Results

### Description of the farmers, goat population, and management practices

Farmer’s age ranged from 18 to 83 years old (mean=47.98 years; median=48 years). Most of them were male (50/81; 61.73%) and single (61.73%) with an average goat raising experience of 7.8 years. The reasons for keeping goats involved buy and sell (70.44%), breeding (50.22%), consumption (45.21%), and leisure (17.11%). On the average, each farmer raised about six goats with a median of three. Goats were derived mainly from the city (71.11%), but other farmers outsourced from Mahaplag (4.62%), Ormoc (10.77%), Abuyog (1.54%), Camotes (13.85%), Davao (56.92), Villaba (4.62%), and Tabango (7.69%).

All of the 450 goats were intended for meat production. As for breed, 169 (37.56%) were mixed breed and 281 (62.44%) were Philippines native. Goat’s age ranged from 1 month to 9 years old (mean=22.46 months; median=18 months). As for goat sex, 143 (31.78%) were male and 307 (68.22%) were female. About 86% of the sampled goats were located in areas with an average elevation of 20.59 m, whereas 13.56% were near the coastal sites. Sampled goats were mainly kept in makeshift goat houses made of scrap woods and bamboos (68.67%). Few goats were kept in concrete houses (19.11%) and under the tree (0.22%). Goat house flooring was either made of wood (48.44%) or cement (30.22%) although 28.89% utilized the ground. Other animals raised along with goats were dogs (49.11%), cats (8.89%), turkeys (6.06%), carabaos (24.24%), ducks (3.03%), pigs (21.21%), and chickens (45.45%). All goats in the herd were kept together, whereas 52.89% of other animal species had an association or in contact with goats. The method of goat feeding mostly involved pasture grazing (97.11%). In addition, 29.11% of farmers offered cut and carry grasses. All farmers fed their goats with a variety of vegetation whenever available in the locality. In particular, they provided Kakawate leaves (*Gliricidia sepium*) (16.44%), Napier grass (*Pennisetum purpureum*) (22.70%), Carabao grass (*Paspalum conjugatum*) (17.08%), Ipil-ipil leaves (*Leucaena leucocephala*) (8.76%), and Trichanthera (*Trichanthera gigantea*) (7.11%).

About 60% of the goat farmers experienced disease occurrence in the form of diarrhea, lameness, and lethargy. Only 144 out of 450 farmers (32%) administered vitamins and mineral supplementation to goats but not in a regular basis. Farmers were also using antibiotics (21.78%) and dewormers (76.44%). They only dewormed goats when there is diarrhea (62%) and before the breeding period (14.44%). Examination of the sampled goats during field visits revealed that 20.63% were anemic (FAMACHA^©^ score 4 to 5), and 20.44% were emaciated with poor hair coat (body condition score below 3 in 1-5 scale) [16,17]. Disease control measures, such as isolation, were conducted according to the 77% of farmers, but these measures were below standard. All farmers were cleaning the goat houses either daily (83.33%) or once a week (16.67%). The method of cleaning involved rinsing plus the use of hard broom (34%) or the use of hard broom alone (66%).

### Prevalence of gastrointestinal tract (GIT) nematodes

The prevalence of GIT nematode mono- and co-infections through fecalysis is presented in Table-1. Fecalysis showed two groups of nematode: Strongyle and whipworm (*Trichuris* spp.). The herd prevalence of strongyle and whipworm infections was 100% and 4.94% (95% CI: 0.12-9.76), respectively. The animal-level prevalence of strongyle infection remained higher at 96.22% (95% CI: 93.90-97.71) than the 4.44% (95% CI: 2.81-6.90) prevalence of whipworm infection. The animal-level prevalence of mixed infection at 4% (95% CI: 2.46-6.37) showed little variation with herd-level prevalence.

**Table-1:** Prevalence of gastrointestinal nematodes in goats in Baybay City, Leyte, through fecalysis.

Parasite	n/N	Prevalence (%)	95% CI
Herd-level prevalence			
Strongyle	81/81	100	-
Whipworm (*Trichuris* spp.)	4/81	4.94	0.12-9.76
Animal-level prevalence			
Strongyle	433/450	96.22	93.90-97.71
Whipworm (*Trichuris* spp.)	20/450	4.44	2.81-6.90
Strongyle+Whipworm	18/450	4.00	2.46-6.37

n/N=Positive goats or herd over the total number of goats or herd. CI=Confidence interval

### EPG count of GIT nematode

Strongyle EPG count in all goats ranged from 0 to 2850 with the mean of 323.04 (95% CI: 290.74-355.35). Meanwhile, the EPG count of *Trichuris* infection ranged from 0 to 240 with the mean of 5.80 (95% CI: 3.02-8.58). All 14 barangays were positive for strongyle infection and only three barangays for *Trichuris* infection. Analysis of the degree of nematode infection based on EPG count showed that 82.89% (95% CI: 79.40-86.39) of goats has mild infection, 15.56% (95% CI: 12.19-18.92) has moderate infection, and only 1.56% (95% CI: 0.41-2.70) has heavy infection.

### Strongyle genus

In total, 1400 L3s identified in 14 barangays revealed five strongyles, namely, *Haemonchus, Trichostrongylus, Oesophagostomum, Cooperia*, and *Chabertia*. *Haemonchus* dominated the majority of barangays and constituted for 34.79% of the overall strongyle population; this proportion was statistically comparable to *Trichostrongylus* (33.29%) (p<0.05). *Oesophagostomum* (24.21%) was one of the three most common genera identified. Moreover, *Cooperia* (6.93%) and *Chabertia* (0.79%) were significantly lower among the strongyle detected.

### Risk factors for strongyle and Trichuris infection

Table-2 summarizes the risk factors unconditionally associated with caprine strongylosis at animal-level. Farm-level analysis was not conducted since all farms were positive for strongyle infection. Initial univariate analysis showed the 12 intrinsic and extrinsic variables crudely associated with strongyle prevalence (p<0.20). The 12 significant variables were simultaneously analyzed in a multivariate logistic regression model following a backward stepwise elimination process to identify the most significant risk factors (p<0.05). The associated risk factors identified for strongyle infection were goat’s age and the type of the goat house flooring. As for age, goats older than 12 months were four times more likely to present high strongyle infection when compared to goats <6 months old (p=0.049). Furthermore, with each month increase in goat’s age the odds of acquiring strongyle infection also increased by 1.07 times (p=0.015). Goat houses with cemented flooring were inversely associated with strongyle infection (odds ratio [OR]=0.27; p=0.013).

**Table-2:** Risk factors identified to be unconditionally associated with strongyle infection in goats raised in Baybay City, Leyte.

Variables	Case/exposed	Prev (%)	Crude OR	95% CI	p value	AOR	95% CI	p value
Goat raising experience	-	-	1.18	0.98-1.40	0.068			
Breeding as production scheme	213/226	94.25	0.30	0.10-0.93	0.037			
Number of raised goats	-	-	0.98	0.97-1.00	0.051			
Goat breed								
Native	275/281	97.86	1.00	-	-			
Mixed	158/169	93.49	0.31	0.12-0.86	0.025			
Goat’s age (months)	-	-	1.07	1.02-1.12	0.010	1.07	1.01-1.12	0.015
<6	127/135	94.07	1.00	-	-	1.00	-	-
6-12	68/74	91.89	0.71	0.28-1.80	0.548	0.59	0.23-1.52	0.363
>12	238/241	98.76	5.00	1.62-15.44	0.019	3.92	1.25-12.28	0.049
Goat housing								
Under the tree	1/1	100.00	25.41	15.65-41.26	0.000			
Makeshift house	303/309	98.06	4.27	1.55-11.80	0.005			
Goat house flooring								
Cement	125/136	91.91	0.22	0.08-0.61	0.004	0.27	0.10-0.75	0.013
Wood	215/218	98.61	4.59	1.30-16.20	0.018			
Using antibiotics	91/98	92.86	0.38	0.14-1.03	0.056			
Using dewormer								
Pyrantel	27/429	93.10	0.26	0.03-1.93	0.187			
Pamoate ivermectin	6/7	85.71	0.12	0.01-1.46	0.095			
Giving dewormer before breeding	68/74	91.89	0.34	0.12-0.95	0.040			
Feeding or accessed to *G. sepium* leaves	68/74	91.89	0.37	0.14-1.06	0.061			
Vitamins and mineral supplementation	135/144	93.75	0.04	0.15-1.07	0.067			

Prev=Prevalence, OR=Odds ratio, CI=Confidence interval, AOR=Adjusted Odds Ratio, *G. sepium*=*Gliricidia sepium*

The risk factors unconditionally associated with *Trichuris* infection in goats are presented in Table-3. There were no significant risk factors found on the farm-level, and thus, no model was created. Of the six intrinsic and extrinsic variables associated with trichuriasis in the univariate analysis (p<0.20), the final multivariate model revealed the two most significant risk factors: Leisure as the production scheme and accessed to or feeding carabao grass (*P. conjugatum*). Goat farms adopting leisure as production scheme were 8.12 times more likely to have *Trichuris* infection (p=0.000). Similarly, goats grazing in carabao grass were 5.52 times more likely to acquire the parasite (p=0.001).

**Table-3:** Risk factors identified to be unconditionally associated with *Trichuris* infection in goats raised in Baybay City, Leyte.

Variable	Case/Exposed	Prev (%)	Crude OR	95% CI	p value	AOR	95% CI	p value
Farmer’s age (years)	-	-	1.04	1.01-1.09	0.006			
Production scheme								
Selling	7/317	2.21	0.21	0.08-0.53	0.001			
Leisure	11/77	14.29	6.74	2.69-16.90	0.000	8.12	3.08-21.32	0.000
Number of goats raised	-	-	0.93	0.86-1.01	0.095			
Goat’s age (months)	-	-	1.02	0.99-1.04	0.061			
<6	6/135	4.44	1.00					
6-12	1/74	1.35	0.29	0.03-2.49	0.262			
>12	13/241	5.39	1.23	0.46-3.30	0.697			
Method of feeding								
Cut and carry	2/131	1.53	0.26	0.06-1.13	0.073			
Feeding or accessed to carabao grass (*P. conjugatum*)	9/77	11.69	4.36	1.74-10.91	0.002	5.52	2.07-14.77	0.001

Prev=Prevalence, OR=Odds ratio, CI=Confidence interval, AOR=Adjusted odds ratio, *P. conjugatum*=*Paspalum conjugatum*

## Discussion

This is the first investigation of prevalence and risk factors associated with gastrointestinal nematode infection in goats raised in rural barangays identified by DA - Baybay City with the highest number of goats. All farms or households in the area have at least one goat infected by strongyle nematode, in which 96.22% of animal-level prevalence was observed. The GI nematodes detected in the study were strongyles (i.e., *Haemonchus, Trichostrongylus, Oesophagostomum, Cooperia*, and *Chabertia*) and whipworm (*Trichuris*), which were similarly identified in goat farms in the Philippines [2,18,19].

The high GIT nematode prevalence corroborates with Ducusin and Faylon [20], who reported the major role of parasitism in impeding goat productivity among Filipino farmers. Other Southeast Asian countries likewise experienced remarkably high burden of goat endoparasitism ranging from 80% to 100% prevalence at the herd and animal levels [21,22]. The high burden of GIT nematode in goats is attributed to the lack of parasite control measures in rural, remote regions of many tropical countries where the climate is favorable for parasite survival and dissemination [23-25].

Goat age and goat house flooring were the most significant risk factors for strongyle infection in the study area. Older goats were more likely to acquire strongyle infection than the young ones, with each month increase in age the adjusted OR of strongylosis increased by 1.07 times. This observation was similar to the study conducted in central and southern regions in Korea [26] and Satun province in Thailand [27]. A possible explanation may involve the increased time of goat exposure with L3s in the contaminated pasture. Therefore, grazing of young and adult animals together with poorly drained land could provide an ideal condition for nematode transmission leading to clinical parasitism [28]. In addition, cement flooring in goat house was found to be a protective variable that decreased the odds of strongyle infection. As observed in the study, herd-health management of goats kept in goat houses with cement type of floor was usually better compared to houses with non-cemented flooring. An example of hygienic practice adopted by goat raisers was the use of soap and water in rinsing the cemented floor aside from the application of broom cleaning. Parasite eggs and larvae survival as a result of inefficient cleaning and suitable microenvironments inside the goat house may lead to new infection or reinfection [29]. Therefore, parasites can be avoided by following the standard design of goat houses which involved building elevation from the ground by 1-1.5 m and adopting a slatted floor type with 1 cm space between to facilitate efficient fecal removal [3]. Actually, elevated and slatted floor goat houses were identified as the significant factors in reducing parasite reinfection in goats [30].

Meanwhile, the most significant risk factors for *Trichuris* infection in goats involved leisure as production scheme and feeding of carabao grass *(P. conjugatum)*. Leisure production increased the odds of trichuriasis since most farmers adopting this type of system were only satisfied with the mere goat presence, and thus has less concern on proper herd-health management. Goats fed with carabao grass are most likely infected with *Trichuris* since this type of grass is usually abundant under coconut plantation and not usually reached by direct sunlight [31]. The presence of suitable microenvironment, i.e., moisture with little sunlight, would mean better parasite’s egg survival [32]. Furthermore, farmers were not practicing rotational grazing resulting in a diminutive height of the grass, thereby increasing the odds of acquiring *Trichuris* eggs in the pasture. In terms of anthelmintic usage, most of the farmers were administering albendazole, and very few were using ivermectin and pyrantel pamoate. Apparently in the study area, the use of anthelmintic did not reduce parasite prevalence in goats, possibly due to incorrect dosage calculation and inappropriate dosing regimen, or due to anthelmintic resistance [18,20].

## Conclusion

Most of the backyard goat raisers in Baybay city, Leyte, were not practicing sound helminth control as suggested by high gastrointestinal nematode prevalence. Based on our results, proper grazing and pasture management, building better goat houses as per standard, and applying strategic deworming or proper usage of anthelmintics must be applied to control goat nematodes and improve goat health and production.

## Authors’ Contributions

APMR and HPP both designed the study and established the workflow. APMR collected the samples in the field, conducted the interview, and performed the fecalysis. HPP supervised APMR in laboratory work, data collection, and data analysis. The first draft was initially written by APMR before being revised by HPP. All authors read and approved the final manuscript.
